# Two-dimensional transition metal dichalcogenides assisted biofunctionalized optical fiber SPR biosensor for efficient and rapid detection of bovine serum albumin

**DOI:** 10.1038/s41598-019-43531-w

**Published:** 2019-05-06

**Authors:** Siddharth Kaushik, Umesh K. Tiwari, Akash Deep, Ravindra K. Sinha

**Affiliations:** 1 CSIR-Central Scientific Instruments Organization, Chandigarh, 160030 India; 2 Academy of Scientific and Innovative Research, CSIR-CSIO Campus, Chandigarh, 160030 India; 3 TIFAC-Centre of Relevance and Excellence in Fiber Optics and Optical Communication, Department of Applied Physics, Delhi Technological University, Delhi, 110042 India

**Keywords:** Two-dimensional materials, Biophotonics

## Abstract

The present study reports an alternative method of functionalizing the optical fiber Surface Plasmon Resonance (SPR) sensing probe with antibodies for label-free detection of bovine serum albumin (BSA) protein. In this novel approach, the gold coated fiber was first modified with Molybdenum disulfide (MoS_2_) nanosheets followed by its bio-functionalization with Anti-BSA antibodies. The developed technique not only allowed the amplification of the SPR signals by synergic effects of MoS_2_ and gold metallic thin film but also enabled a direct and chemical-free attachment of representative antibodies through hydrophobic interactions. The sensitivity of the MoS_2_ modified sensing probe with detection limit of 0.29 µg/mL was improved as compared to the fiber optic SPR biosensor without MoS_2_ overlayer (Detection limit  for BSA was 0.45 μg/mL). The developed biosensor has good specificity, and environmental stability. Accordingly, the proposed design of the MoS_2_ based SPR optical biosensor can offer the development of a simplified optical device for the monitoring of various biomedical and environmental parameters.

## Introduction

Biosensors have been the focus of cutting-edge research in recent years as they possess desirable properties of high sensitivity, excellent specificity, good stability, easy operation, fast response and cost-effective analysis^1^. Considering these imperative features, a plethora of biosensing platforms including photoelectrochemical^2–5^, impedance-based^6–8^ and optical^9,10^, were reported for sensitive and specific detection of chemical compounds and biomolecules. As diseases caused by pathogens including diarrhea^11^, typhoid^12^ and pneumonia^13,14^ etc., are major public health concern in developing nations, a sensitive and portable biosensor is needed to rapidly detect the target analyte in a different matrices without delayed intermediate sample processing. SPR sensors offer a specific, rapid, sensitive and label-free detection method preferable for the medical diagnostics and chemical analyses^15^. For the last three decades, since its inception in 1982 as a gas sensor^16^, the Surface Plasmon Resonance (SPR) sensors have evolved as the propitious sensing platform for wide range of applications^17–22^. For medical and environmental applications different SPR based configurations had been explored which includes prism-based SPR sensor^23,24^, optical fiber SPR sensors^25–28^.

The prism configuration has certain limitations including large size with many optical and mechanical components which limit their use for on-site sensing application^29^. The optical fiber-based sensors have the potential to be used for the on-site sensing of analytes as they allow the designing of devices with advantageous features of compactness, cost-effectiveness, robustness, and rapid response^30–34^. An easier tuning of the resonance wavelength in optical fiber SPR sensors compared to the prism-based SPR sensors is also of great benefit^35,36^. As such, the resonance characteristics depend upon the core refractive index (RI) and RI of the surrounding layer, metal layer thickness, and the length of the sensing probe^37–40^. Additionally, the optical fiber sensors are immune to electromagnetic interference and undesired influences of ionization radiation^41–43^. Hence the fiber optic SPR sensors are practically advantageous as compared to the other optical sensors in different fields of sensing.

The electromagnetic waves excited by the charge density oscillations that exist along the metal–dielectric interface is known as surface plasmon polaritons (SPPs)^44^. SPR is the quantum of these oscillations and the wave associated with it is called surface plasmon wave (SPW). The SPWs which propagates along the interface are transverse in nature and exponentially decay in both the dielectric and metal medium. TM polarized light excite the surface plasmon at the phase matching condition. As the propagation constant of the TM polarized light matches with the SP wave propagation constant, it results in the generation of surface plasmon resonance (SPR). The normalized transmission power of the fiber optic SPR sensor is presented in eq. (1)^45^

1 $$T.P.=\frac{{\int }_{{\varnothing }_{cr}}^{\frac{\pi }{2}}{R}^{N(\varnothing )}\frac{{{n}_{1}}^{2}Sin2\varnothing }{2\,{(1-{{n}_{1}}^{2}Co{s}^{2}\varnothing )}^{2}}d\varnothing }{{\int }_{{\varnothing }_{cr}}^{\frac{\pi }{2}}\frac{{{n}_{1}}^{2}Sin2\varnothing }{2\,{(1-{{n}_{1}}^{2}Co{s}^{2}\varnothing )}^{2}}d\varnothing }$$

Where n_1_ is the RI of the core and $\varnothing$ is the angle of the incidence of the light with the normal at the core-cladding interface in the sensing region. The total reflection by the incident light is denoted by *N (ø)*. The field intensity decays exponentially with the formation of SPR exhibiting a sharp dip of SP wave in the spectral response of the transmitted light. Hence, the variations in the RI at the surface of the sensing platform within the evanescent wave region due to the interaction between immobilized bioreceptor (e.g., aptamers, antibodies, lectins, etc.) and target molecules in samples may shift the resonant wavelength significantly^46^.

The optical fiber SPR sensing technology is also beneficial for the detection of biomolecules. To frame the optical fiber biosensor specific towards desired analytes, they must be functionalized by bio-recognition molecules, such as proteins, RNA, DNA, cells, etc. This adherence of the biomolecules over the optical fiber surface is generally achieved via some chemical linkers ((3-Aminopropyl) triethoxysilane, N-Succinimidyl 4-Maleimidobutyrate, etc^47–49^. Most of the above-mentioned surface modifications approaches are tedious and do not necessarily provide a homogenous bio coating.

Recently, the 2D transition metal dichalcogenides (TMDs), particularly MoS_2_, have attracted significant attention of researchers in different scientific fields, due to their high electron conductivity, tuneable band gap, and high optical absorption efficiency^50–53^. The hydrophobic nature, large surface area and presence of free sulphur atoms are distinctive features of MoS_2_ which makes it a potential material to develop biosensing interfaces^54,55^. Moreover, the MoS_2_ layers have been employed to inhibit the oxidation of metallic layers such as aluminium in SPR sensors^56^.

The present research outlines a novel method for biofunctionalization and sensitivity enhancement of the optical fiber SPR sensor for biosensing applications. The interfacing of MoS_2_ sheets facilitates simple and rapid antibodies immobilization on transducer’s surface via a convenient method of hydrophobic interaction^57^. In this way, the developed sensor avoids the requirement of any harsh chemical or muti-step treatments to the fiber which affects the sensitivity of the sensor by forming additional layer between antibodies and sensing probe.

## Materials and Methods

### Reagents

Sodium sulfide (Na_2_S), Poly (ethylene glycol) (PEG), Fluorescein isothiocyanate (FITC), 3-mercaptopropionic acid, n-methyl pyrrolidone (NMP) was purchased from Sigma-Aldrich, India. Umicore, India supplied molybdenum sheets (99.5% purity), Hydrofluoric acid (48% w/w), Anti-Bovine serum albumin (monoclonal), Bovine serum albumin and 10 mM Phosphate buffer saline (pH 7.4 at 25 °C) were obtained from Merck, India. All the other chemicals used were of the analytical grade. All the solutions were prepared in double deionized water >18 mΩ.cm (specific resistivity) at 25 °C, Millipore.

### Analytical instruments

The optical absorbance spectrum of the MoS_2_ functionalized optical fiber sensor was recorded at a scan rate of 600 nm min^−1^ over a wavelength range of 200–800 nm by a UV-Vis spectrophotometer (Varian Cary 5000, Agilent Technologies, U.S.A). The X-Ray diffraction analysis was achieved with a Bruker D8 diffractometer using Cu Kα radiation (wavelength = 1.546 Å) at the scan rate of 4° min^−1^. The morphological characterization of the fabricated biosensor was performed by field-emission scanning electron microscope (Hitachi S4800; accelerating voltage 2–4 KV). The Raman analysis was performed using a Raman spectrophotometer (excitation wavelength: 514 nm) (Renishaw, UK). The confocal laser scanning microscopic (LSM 510) (Zeiss, Germany) was used for morphological analysis of the bio functionalized sensing probe. The exfoliated MoS_2_ nanosheets were analysed using a transmission electron microscope (TEM) (Applied voltage: 200 kV) (JEOL 2010, Japan).

### Experimental setup

**Figure 1:**
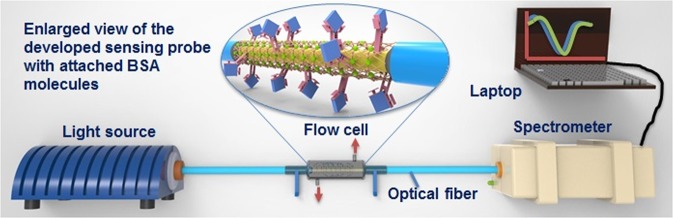
Diagrammatic representation of the SPR biosensor experimental system for the detection of BSA. The time of interaction between Anti-BSA antibodies and BSA sample was 15 minutes.

The experimental setup employed to study the SPR biosensor performance is presented in Fig. 1. The developed sensor (Anti-BSA/MoS_2_/Au optical fiber) was positioned in a glass flow cell having definite inlet and outlet passages. Non-polarized polychromatic light from broadband source (wavelength range: 360–2400 nm; output power: 6.7 mW; Ocean Optics, HL-2000,) was launched at one end of the fiber optic sensor. The transmission spectra were recorded through charge-coupled device (CCD) spectrometer (λ: 200–1160 nm; resolution: 0.09–20 nm; Avantes, ULS2048XL-EVO) connected to another end of the sensor. The computer was interfaced with the spectrometer to record the spectral response at different concentrations of the BSA.

### Synthesis of MoS_2_ nanosheets

MoS_2_ nanosheets were synthesized by the electro-dissolution method. Briefly, the cathode having molybdenum sheet was intercalated with Na^+^ ions. The Ag/AgCl, molybdenum sheets and Pt wire were forgathered in an electrochemical cell as the reference, cathode (working) and anode (counter) electrodes respectively. 1 M sodium sulfide (Na_2_S) solution was taken as the electrolyte. The chrono-amperometry mode was applied, keeping the potential constant at 1 V. The electrolysis process was repeated for multiple cycles to obtain better product yield. The hydroxyl and oxygen radicals generated by the oxidation of water react with the grain boundaries and edge sites of the molybdenum sheets creating defective sites. The Na^+^ ions intercalate in these defective sites causing interlayer separation. The Na^+^ intercalated MoS_2_ sheets dissolution was clear, as the colorless electrolyte organic solution transformed primarily to pale yellow and ultimately to dark yellow colour with the trickling of MoS_2_ sheets into the solution. The extracted material was separated by centrifugation at 20000 rpm for 30 min. After washing three times with DI water followed by centrifugation, the supernatant was suspended in DI water and ultrasonicated for 2 hours resulting in the exfoliation of Mo into few-layered MoS_2_ nanosheets. This method is rapid and highly efficient for producing MoS_2_ nanosheets.

### Fabrication of the sensing probe

#### Chemical etching of multimode optical fiber

The optical fiber SPR sensor was fabricated in a plastic cladded multimode step-index fiber (core diameter: 400 μm). For developing the sensing region, cladding layer was initially removed by thermal process to expose a 1 cm uncladded fiber length. After depletion of the cladding layer, the core region of the optical fiber was subjected to the chemical etching process. It was performed by exposing the fiber to Hydrofluoric acid (HF) for 38 minutes at room temperature (25 °C ± 2 °C). The acid solution was intermittently agitated to allow a uniform etching. The etching reaction proceeded at an etch rate of 3 microns/minute. Subsequently, the etched fiber was washed with methanol and DI water. The tapered optical fibers were stored in glass desiccator under dry conditions.

#### DC Magnetron sputtering unit assisted gold thin film deposition on the optical fiber (PVD method)

The gold thin film coating on the tapered optical fiber was achieved through a DC Magnetron Sputtering deposition instrument which consisted of a DC sputter source (Excel Instruments, India). The optical fiber was placed at a distance of 10 cm in front of the target. A glow discharge was created by applying high voltage. The acceleration of argon ions to a pre-mounted gold target surface led to the ejection of gold particles, which subsequently got adhered as a sputtered coating layer onto the optical fiber. During the above experiment, the sputtering gas (Argon) was introduced in the chamber with a flow rate of 30 standard cubic centimetre per minute (SCCM) at a pressure of 2.1 Pa. To achieve good sensitivity, the gold layer thickness is considered as an important parameter. The fiber was rotated for different cycles (4, 5, 6, 7, 8, 9) to optimize the gold layer deposition to achieve best results and it was observed that after 7 cycles, the sensitivity of the fabricated sensor towards 6% w/v sucrose solution (RI: 1.3420 at 25 °C) was highest as compared to the other rotations of certain time interval as presented in Fig. S1(a) (Supplementary Information). To introduce a uniform coating, 7 cycles of rotation was done under the inert conditions. The chromium (~5 nm) was initially coated on the optical fiber for good adhesion of gold on optical fiber. The DC power of 40 W was used during the total deposition time. The thickness of the coated gold was 50 nm ± 4 nm which is comparable to the thickness reported in preceding studies^58^.

### Functionalizing the MoS_2_ nanosheets on the gold coated optical fiber

The MoS_2_ overlayer were deposited on the gold coated fiber optic SPR sensor via dip coating technique. The gold coated fiber was incubated with 1 mL solution of the MoS_2_ nanosheets. To optimize the nanosheets functionalization process the dip coating was done for 2, 3, 4, 5, 6, 7 cycles. Each cycle was accomplished by dipping the optical fiber in the MoS_2_ nanosheets solution for 30 seconds and subsequently drying for 120 seconds. After different cycles, the MoS_2_ functionalized sensing probe was annealed for 1 hour at 60 °C to confirm the proper robustness of the MoS_2_ interfacing with the gold layer. The MoS_2_ functionalized sensing probes were further treated with Anti-BSA antibodies and the biofunctionalized sensing probes were tested for detection of BSA (10 µg/mL). The dip coating process of 05 cycles was selected for the development of proposed sensor as best resonance wavelength shift was obtained at this optimal thickness of MoS_2_ layers as depicted in Fig. S1(b) (Supplementary Information). The Raman spectroscopy and scanning electron microscopy characterization confirmed the uniform interfacing of the MoS_2_ on the gold coated fiber.

In SPR based sensing, temperature is an important parameter which affects metal properties through phonon-electron scattering and electron-electron scattering, signal-to-noise ratio (SNR) and sensitivity of the sensor^59^. The metal dielectric function is also related with temperature variation. It has been reported that plasma frequency and collision frequency of metal layer is related to temperature, the RI of core and RI of the sensing layer are also affected by temperature through thermo-optic coefficient. The blue shift in resonance wavelength and broadening of SPR curve was noted with increase in temperature in previous studies^60^. We have performed our experiments at room temperature (25 °C ± 2 °C) to eliminate the temperature effect on spectral response of the proposed sensing platform.

### Biofunctionalization of Anti-BSA on MoS_2_/gold/optical fiber

The MoS_2_ functionalized surface of the optical fiber was incubated with the 80 µg/mL solution of anti-BSA (monoclonal antibodies) in PBS solution (pH 7.4) for two hours at room temperature. After immobilization of anti-BSA antibodies, the sensing platform was incubated for 20 minutes with 35 μL of 1-mM polyethylene glycol solution as a blocking agent to occlude the non-specific sites of the sensing probe. After an incubation, the fiber was washed three times with DI water for removal of unbound molecules.

### Sample preparation for characterization of the bio functionalized sensing probe

We have immobilized the FITC conjugated anti-bovine serum albumin antibodies on MoS_2_/Au/optical fiber through simple physical adsorption route. Briefly, 500 µL of FITC (0.2 mg/mL prepared in DMF) and 80 µg/mL of anti-*BSA* antibodies were mixed together. The mixed solution was incubated for 1 hour in dark at 25 °C. Desalting spin column was used to remove excess FITC molecules. The 1 mg/mL solution of FITC/anti-BSA in PBS solution (pH 7.4) was prepared and then left to incubate with the MoS_2_ functionalized surface of the optical fiber for 40 min at room temperature. For the Raman spectroscopic analysis, the MoS_2_/Au/optical fiber was first carefully diced using a surgical blade, then mounted on the glass slide. A 50X objective was employed to focus the laser beam (514 nm) and collect Raman signal at room temperature.

### Quantitative analysis of Bovine serum albumin (BSA)

**Figure 2:**
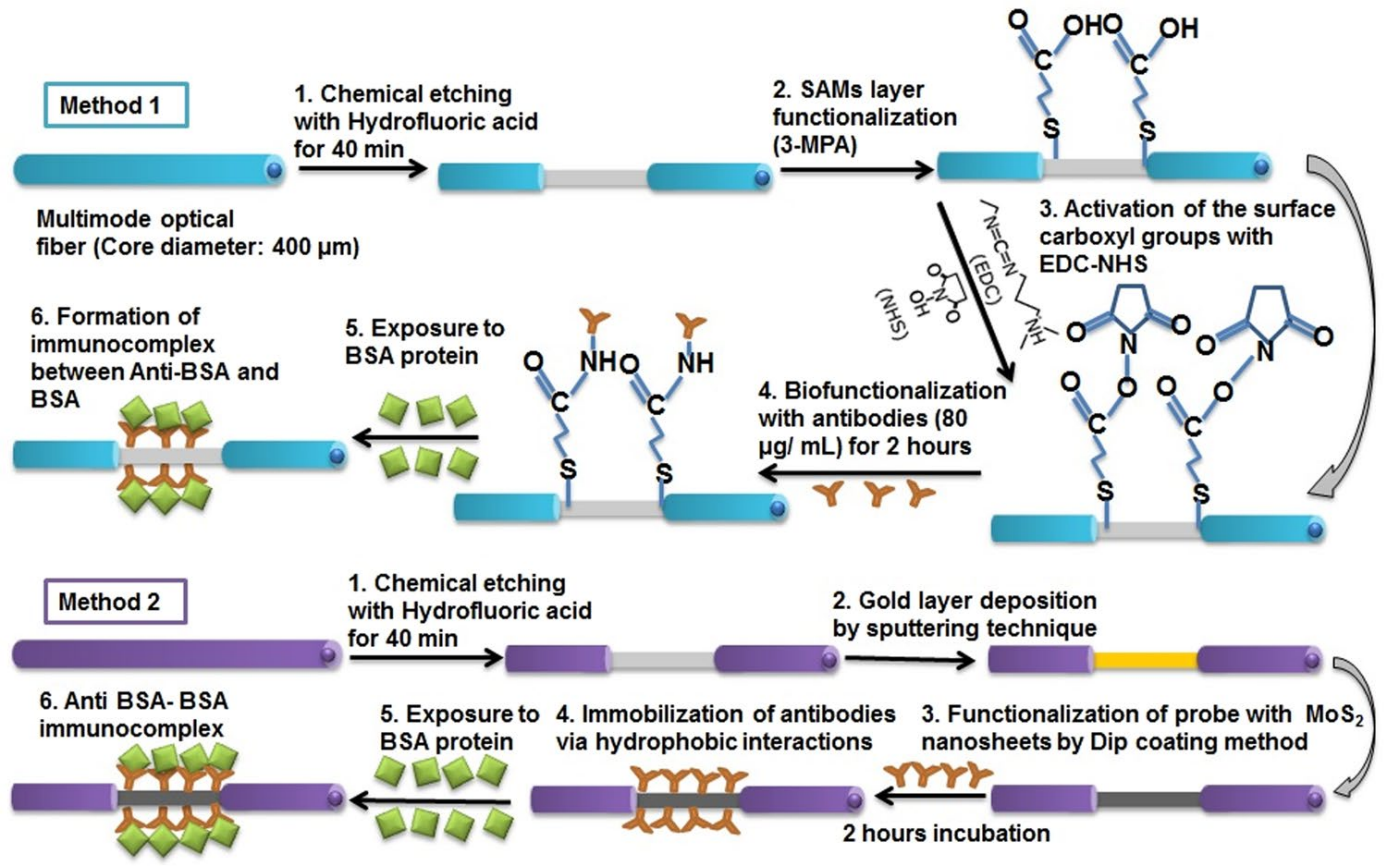
Schematic delineation of the development process of optical fiber SPR biosensor without MoS_2_ overlayer and the MoS_2_ modified optical fiber SPR biosensor.

The performance of the antibody immobilized optical fiber sensor with MoS_2_ overlayer was collated with antibody immobilized gold coated optical fiber sensor without MoS_2_ overlayer. In this conventional design, the gold coated optical fiber was modified with 3-mercaptopropionic acid (3-MPA) by immersing the gold coated fiber in 10 mM ethanolic solution of 3-MPA for 6 hours. It was then incubated with 1 mL of PBS solution having 1 mM EDC and 2 mM NHS to activate –COOH groups of the sensing probe. Incubation of the anti-BSA antibodies was finally performed at 37 °C for an hour. The remaining binding sites on the sensing probe were blocked by treating the developed sensor with with 1 M ethanolamine hydrochloride (pH 8.6) for 45 min. The developed biosensor was then thoroughly washed with distilled water three times, dried by nitrogen gas, and stored at 4 °C before using for the BSA detection. The complete process for fabrication of SPR biosensor without MoS_2_ overlayer and MoS_2_ assisted biofunctionalized SPR sensing probe is presented in Fig. 2. Different concentration of BSA, ranging from 10 μg/mL to 50 μg/mL in PBS solution was prepared. These samples were then inserted through flow cell to interact with the antibody immobilized MoS_2_/gold/optical fiber sensor for 15 minutes and then the resulting transmission spectra were recorded. The spectral response in terms of shift in resonance wavelength of the sensor was then correlated with the defined concentrations of BSA.

## Results and Discussion

### Structural analysis of exfoliated MoS_2_ nanosheets and developed optical fiber SPR sensor

**Figure 3:**
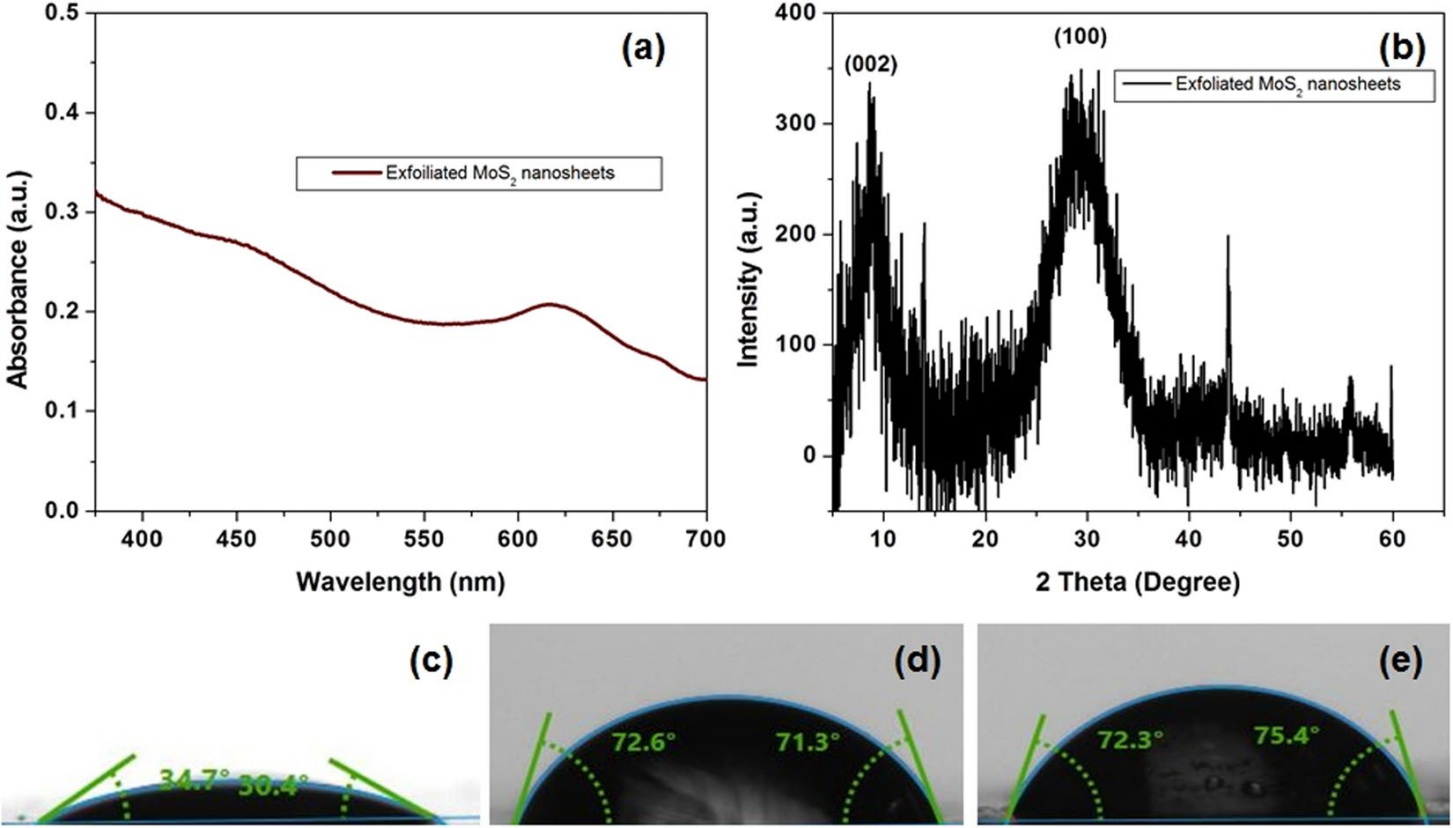
**(a)** UV-Vis spectra of exfoliated MoS_2_; **(b)** XRD analysis of exfoliated MoS_2_ nanosheets; The water contact angle of **(c)** silicon substrate (SiO_2_) **(d)** gold (Au) and **(e)** MoS_2_ layer to determine the hydrophobic characteristics of different substrates. The mean water contact angle of MoS_2_, Au, and SiO_2_ substrate are 32.55°, 71.95°, and 73.85° respectively.

The exfoliated MoS_2_ nanosheets have been characterized by well-established microscopic and spectroscopic techniques. In UV-Vis absorption spectrum [Fig. 3(a)], the exfoliated MoS_2_ nanosheets have shown the absorption peaks at 617 nm and 672 nm, due to direct transitions at the K point of the Brillion zone^61^. The broad peak at 395 around 453 nm originating after the linear transition of electrons from deep valence band to the conduction band are noted as mentioned in the reported studies^62^.

In XRD analysis of the bulk MoS_2_ powder many peaks are evident owing to the lattice plane reflections from the multiple layers of MoS_2_ [Fig. 3(b)]. However, the XRD spectra of  exfoliated MoS_2_ nanosheets shows the strong distinctive (002) peak of bulk MoS_2_ (at around 14° was shifted to 9°) signifying lattice expansion. The shift is due to an increased lattice strain and reduction in crystallite size. The exfoliation causes increase in the interlayer distance of the (002) lattice plane thereby changing the diffraction at a lower angle. The manifestation of XRD peaks at 33° corresponds with planes of (100) as per the typical hexagonal MoS_2_ structure. The broadening of these peaks are correlated with the reduction of particle size in the corresponding planes^63^.

To determine the hydrophobicity of the exfoliated MoS_2_ nanosheets, drop shape analysis (DSA) was performed. The contact angle was measured by the image of sessile water drop at the points of contact with the target surface as shown in Fig. 3(c–e). The glass substrate is hydrophilic in nature with mean contact angle of 32.55°. The mean contact angle of gold coated surface (71.95°) is relatively less hydrophobic as compared to MoS_2_ nanosheets functionalized surface (73.85°). The higher hydrophobicity of MoS_2_ nanosheets helps in proper adsorption of proteins and antibodies without any cross likers. Hence, MoS_2_ nanosheets are potential 2 D nanomaterials for rapid and simple immobilization of antibodies.

**Figure 4:**
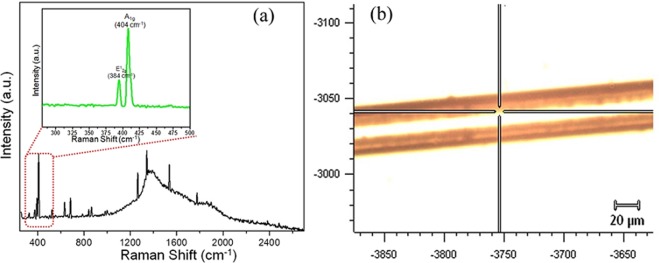
Raman analysis of the MoS_2_ nanosheets functionalized sensing probe **(a)** Characteristic in-plane E^1^_2g_ (∼384 cm^−1^) and out-of-plane A_1g_ (∼404 cm^−1^) Raman vibrational modes of exfoliated MoS_2_; **(b)** Point of analysis on sensing probe.

Raman spectroscopy is an important technique used to determine the layers of the 2D materials. The peak position and intensity of in-plane (E^1^_2g_) and out-of-plane (A_1g_) modes offers specific identification of discrete and few layers of MoS_2_^64,65^. The E^1^_2g_ (384 cm^−1^) peaks and A_1g_ (404 cm^−1^) peaks were observed in Fig. 4, in accordance with the previous studies^66^. The red shift in the E^1^_2g_ peaks and the blue shift in the A_1g_ peaks were observed indicating decrease in layer thickness from bulk MoS_2_ to exfoliated MoS_2_.

**Figure 5:**
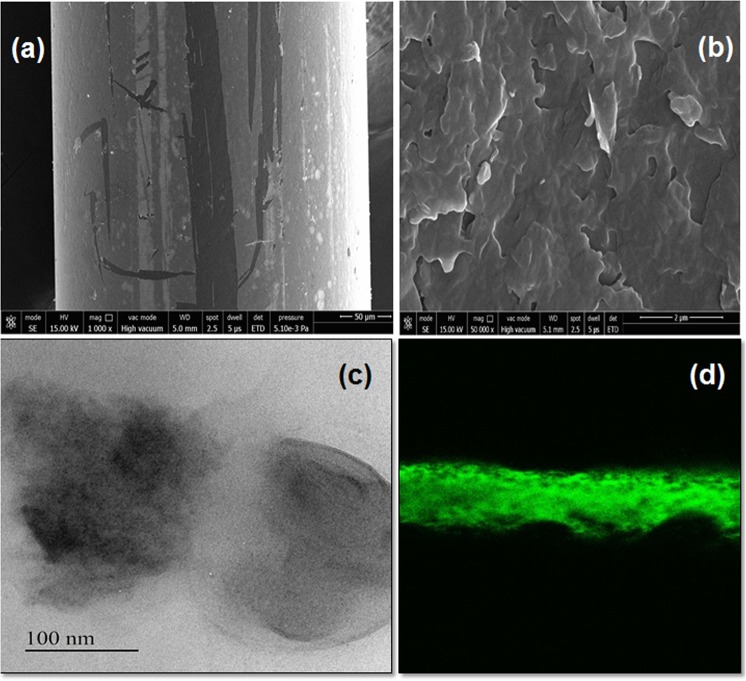
FESEM analysis of **(a)** developed SPR sensor scratched at different points to show the functionalized layers of MoS_2_ nanosheets on the gold coated optical fiber; **(b)** Enlarged view of functionalized MoS_2_ nanosheets; **(c)** TEM analysis of the exfoliated MoS_2_ nanosheets; **(d)** Confocal image of the Anti-BSA monoclonal antibodies immobilized MoS_2_/Au/optical fiber.

The microscopic analysis as shown in Fig. 5(a,b) signifies morphological changes on the surface of sensing probe after MoS_2_ functionalization. It can be observed that the dip coating method explored for the nanosheets functionalization aid in uniform deposition of MoS_2_ on the optical fiber. The FESEM images confirm the homogenous interfacing of MoS_2_ nanosheets, thereby providing higher antibodies binding sites as compared to the fiber optic SPR sensor without MoS_2_ nanosheets.

The formation of the nanostructures with uniform dimensions is evident from the TEM images is presented in Fig. 5(c). The sheets like morphology of the exfoliated MoS_2_ are well demonstrated by the TEM image. The uniform fluorescence signals originating from the FITC/anti-BSA antibodies/MoS_2_/Au/optical fiber as depicted in Fig. 5(d) of confocal laser scanning microscope (CLSM) study affirm the successful immobilization of anti-BSA antibodies over MoS_2_/Au/optical fiber. As a control experiment, sensing platform without bioreceptors (antibodies) under the similar conditions was also observed by CLSM and no fluorescent signals were detected. Thus, the fluorescence perceived in the sensing probe can be attributed to the immobilization of Anti-BSA monoclonal antibodies.

### Comparative study of spectral response characteristics

**Figure 6:**
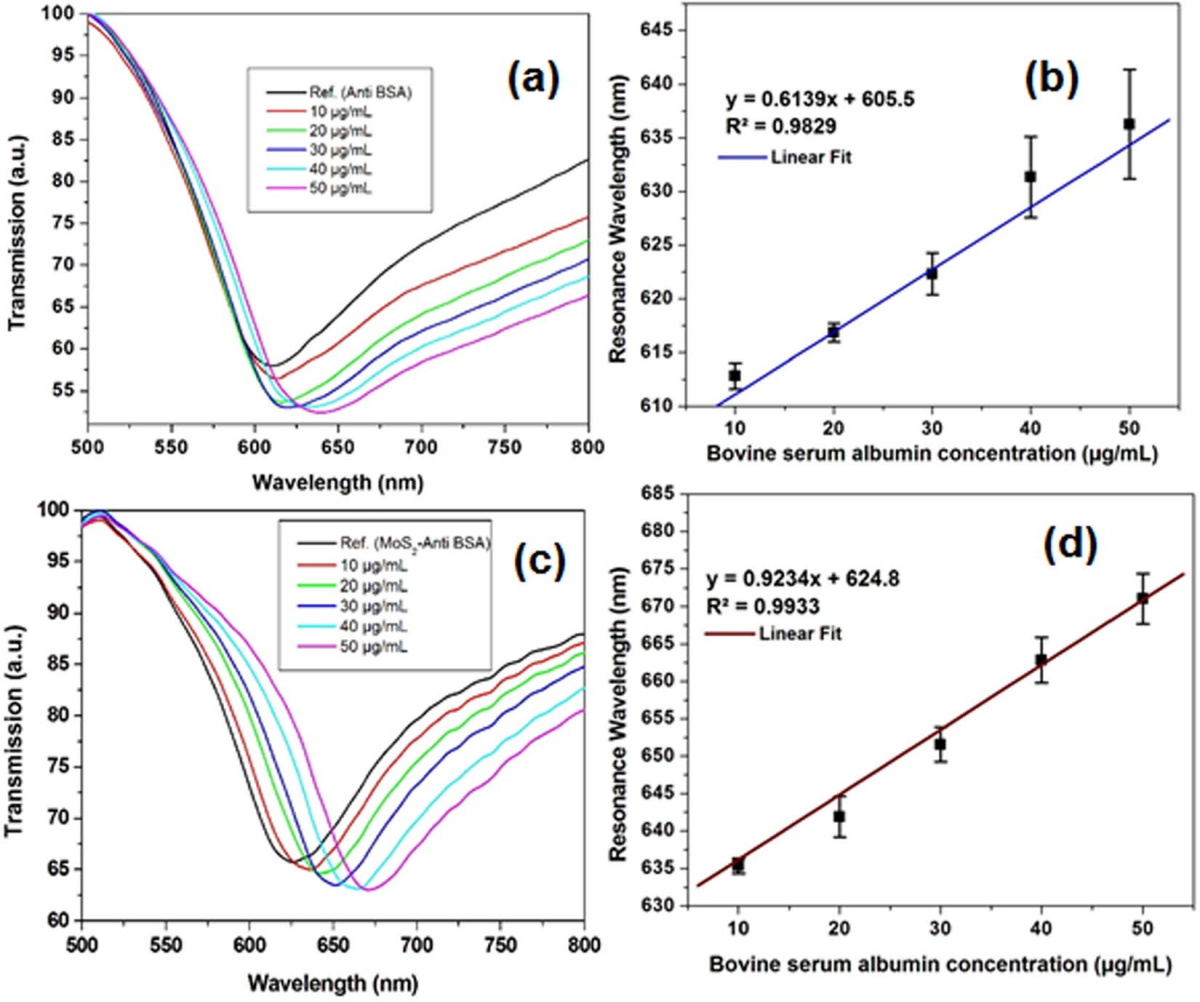
Spectral characteristics towards BSA in PBS solution of the **(a)** optical fiber SPR biosensor without MoS_2_ overlayer and **(c)** developed SPR biosensor; Standard calibration plot of **(b)** Ab/gold/fiber; **(d)** Ab/MoS_2_/gold/fiber against varying concentration of BSA in PBS solution (pH 7.4); The error bars represent the standard deviation, Experiment for each sample was repeated three times (n = 3).

To investigate the output characteristics of the developed SPR biosensor, spectral response was measured for known concentrations of BSA in buffer solution with an Ab/Au/optical fiber (SPR biosensor without MoS_2_ overlayer) and Ab/MoS_2_/Au/optical fiber (developed SPR biosensor). The resonance wavelength observed after the immobilization of the antibodies was regarded as reference peak. The SPR spectra attained with SPR biosensor without MoS_2_ overlayer for different concentration of BSA ranging from 10 µg/mL to 50 µg/mL in PBS solution is depicted in Fig. 6(a). The transmission spectra were measured after 15 minutes of analyte addition in the glass flow cell. For each sample with certain concentration, resonance wavelength was measured. As the concentration of the BSA increases, red shift in the resonance wavelength can be observed. Figure 6(c) shows the respective shift in the resonance wavelength with each sample (BSA) by developed biosensor. The error bar graph shown in Fig. 6(b,d) clearly shows the increase in the resonance wavelength increases linearly with the BSA concentration in PBS solution by Ab/Au/fiber, in PBS solution by Ab/MoS_2_/Au/ fiber. Experiment for the analysis of individual sample was done in triplicates. The probe was treated with DI water for removal of bonded molecules after each experiment. The shift in the resonance wavelength was because BSA and Anti-BSA antibodies interaction results in the formation of immune-complex which changes the surrounding refractive index and modifies the properties of the interacting evanescent wave. The concentration of the BSA protein is directly proportional to the change in the refractive index and the difference in refractive index is marked by the shift in resonance wavelength of the transmission spectra. The level of wavelength shift is higher in the proposed Ab/MoS_2_/Au/optical fiber demonstrating better sensor sensitivity as shown in Fig. S2 (Supplementary Information). Due to high surface to volume ratio of functionalized MoS_2_ nanosheets, the density of functionalized antibodies increases leading to binding of large number of target analytes (*BSA*). It is also important to note that the MoS_2_ single layer has higher refractive index (4.49 @ λ = 651 nm) as compared to gold layer^67^. Due to large RI, the MoS_2_ nanosheets can sustain evanescent waves even at smaller thicknesses. Accordingly, high proportion of evanescent field can be propagated in the sensing region by thin layers (8 ± 2 µm) of these nanosheets. Similarly, as the sensitivity is related to the electrical intensity’s overlap integral in the surrounding medium which depends on the interaction between analytes and guided wave, the greater interaction of analytes and guided waves increases the sensitivity of the immunosensor. It was noted that the proposed SPR biosensor provide better sensitivity with LOD of 0.29 µg/mL [(calculated by the slope of the regression line equation) with R^2 = ^0.9933] than conventional fiber optic SPR biosensor without MoS_2_ overlayer (0.45 µg/mL with R^2^ = 0.9829).

Researchers have reported graphene oxide (GO) functionalized biosensor for the selective detection of BSA (detection limit: 265 μg/mL). This aggregation-induced emission (AIE) based sensing platform utilizes disulfonated tetraphenylethene (TPE-SO3Na) and GO for the detection of BSA molecules^68^. Similarly, quantum dots based fluorescent probes were studied for BSA detection. The binding of BSA to the sensing probe resulted in photoluminescence quenching. The limit of detection of this sensing method was 100 μg/mL^69^. In recent years, the fiber optic sensing platforms have been studied for the detection of BSA. A fiber optic Mach-Zehnder interferometer based BSA detection has been reported with the detection limit of 0.257 μg/mL. The sensing platform was highly sensitive nevertheless, the specificity of the developed sensor was not reported^70^. Nayak *et al*., 2017 have studied the fiber optic biosensor coated with gold nanoparticles and graphene oxide (GO) for BSA detection. This sensing probe functionalized with nanoparticles was studied for detection of 10 μg/mL of BSA^71^. In comparison to the previous studies for the BSA detection, the present work encompassing MoS_2_ nanosheets provide label-free, specific and rapid detection of BSA with comparable sensitivity (detection limit: 0.29 µg/mL). The addition of MoS_2_ nanosheets in the transducer layer increases the light absorption in the sensing medium leading to enhancement of the evanescent field, thereby improving the performance of fiber optic SPR sensor. Though the sensor has better sensitivity towards target analyte (BSA) as compared to the reported fiber optics biosensors, the sensitivity of electrochemical biosensor^72^, differential pulse voltammogram^73^, cyclic voltammetry based sensor^74^, Fluorescence based assay and resonance light scattering spectra (RLS) based assay^75^ is higher as compared to the developed sensor reported in the present study.

### Specificity studies

**Figure 7:**
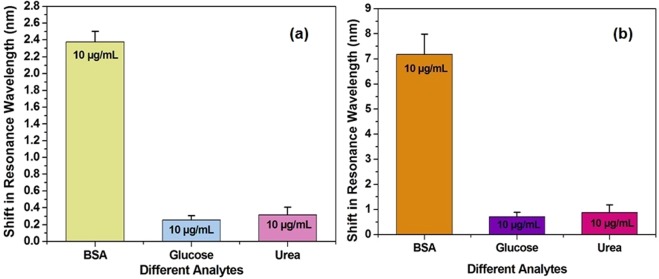
Comparison of specificity **(a)** optical fiber SPR biosensor without MoS_2_ overlayer and **(b)** developed optical fiber SPR biosensor with MoS_2_ overlayer for different compounds in PBS solution.

The specificity towards target analyte is an essential feature of good biosensor. To study the specificity of both the SPR biosensor without MoS_2_ overlayer and the developed MoS_2_ modified SPR biosensor, the spectral analysis was done for cross-reactants like glucose (10 µg/mL) and urea (10 µg/mL). The measured response of both the sensing probes expressed as shift in resonance wavelength towards each compound is shown in Fig. 7(a,b). Comparison of the performance of the developed biosensor with the SPR biosensor without MoS_2_ overlayer indicates that the MoS_2_ based developed biosensor is more sensitive and specific towards the detection of BSA.

### Statistical analysis

To study the significant difference in the mean wavelengths statistically, One-way analysis of variance (ANOVA) was used. The ANOVA test indicate a statistically substantial difference in the resonant wavelength across at least one of the analyte sample (F 3, 8 = 1662.612, p < 0.001), obtained using the MoS_2_ modified sensor. To perform pair-wise comparisons while controlling the family-wise alpha to 0.05 the Tukey’s Honest Significant Difference (HSD) post hoc test was used. Mean wavelength in the detection of BSA observed to be significantly different from that of other analyte including glucose (p < 0.001), urea (p < 0.001), and blank sample (p < 0.001). When compared to the blank sample, no significant difference in mean wavelength in the detection of glucose (p = 0.095) and urea (p = 0.547) was observed. Table S1 (Supplementary Information) presents the pair wise comparison of mean resonance wavelength across different samples. The statistical test was performed by SPSS software version 15.0 (SPSS South Asia, Bangalore). The results confirm the high specificity of the developed biosensor for BSA detection.

## Conclusion

In summary, MoS_2_ nanosheets assisted biofunctionalized optical fiber SPR sensor has been successfully demonstrated for specific detection of BSA protein. The sensing probe functionalization with nanosheets and antibodies was characterized by different analytical techniques including Raman spectroscopy, TEM, UV-Vis spectroscopy, FESEM, confocal imaging and XRD analysis. The optimization of the parameters in the development of sensing probe was performed to attain the good analytical performance. The probe was introduced for the concentration range of 10 µg/mL to 50 µg/mL. The sensing capabilities of the developed biosensor was compared with the fiber optic SPR biosensor without MoS_2_ overlayer. The senstivity of the designed probe (LOD: 0.29 µg/mL) was better as compared to the optical fiber SPR biosensor without MoS_2_ overlayer (LOD: 0.45 µg/mL). The developed sensing probe was highly specific towards target analyte (BSA) even in the presence of cross-reactive compounds. Though the present study has presented some promising results, few limitations require attention including fragile nature of optical fiber and time consuming gold coating process. The alternative method of simple biofunctionalization may open new horizons towards the development of new generation optical fiber sensor with advantage of fabrication simplicity while offering real time monitoring and ultra-low quantitative analysis.

## Supplementary information

Supplementary Information

## Data Availability

All data analyzed during this study are included in form of graphs in this article (and its Supplementary Information).
