# Supplementary material for: Two-dimensional transition metal dichalcogenides assisted biofunctionalized optical fiber SPR biosensor for efficient and rapid detection of bovine serum albumin
Source: Sci Rep. 2019 May 6;9:6987. doi: 10.1038/s41598-019-43531-w (PMC6502831; doi:10.1038/s41598-019-43531-w)
Supplement: Supplementary file 1 — Supplementary Information [file 41598_2019_43531_MOESM1_ESM.docx]

**Supplementary Information**

**Two-dimensional transition metal dichalcogenides assisted biofunctionalized optical fiber SPR biosensor for efficient and rapid detection of** **bovine serum albumin**

Siddharth Kaushik ^a, b^, Umesh K. Tiwari ^a, b, *^, Akash Deep ^a, b^, Ravindra K. Sinha^a, c^

^a^ Advanced Materials and Sensors (V 4), CSIR-Central Scientific Instruments Organization, Chandigarh-160030, India

^b^ Academy of Scientific and Innovative Research, CSIR-CSIO Campus, Chandigarh-160030, India

^c^ TIFAC-Centre of Relevance and Excellence in Fiber Optics and Optical Communication, Department of Applied Physics, Delhi Technological University, Delhi-110042, India

*Corresponding author email: uktiwari_2003@yahoo.co.in


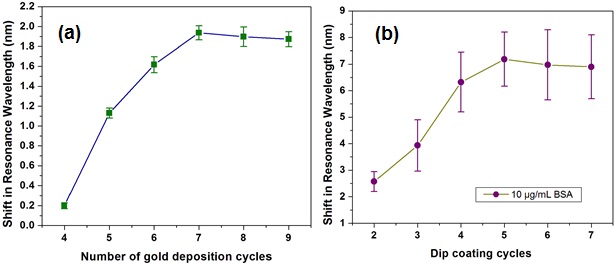


**Fig. S1**. Optimization graphs displaying the (a) relationship between sensitivity and gold layer thickness. The optimum sensitivity was achieved at 50 nm ± 4 nm thickness (7 cycles); **(b)** effect of MoS_2_ layer thickness on spectral response for detection of definite concentration of BSA


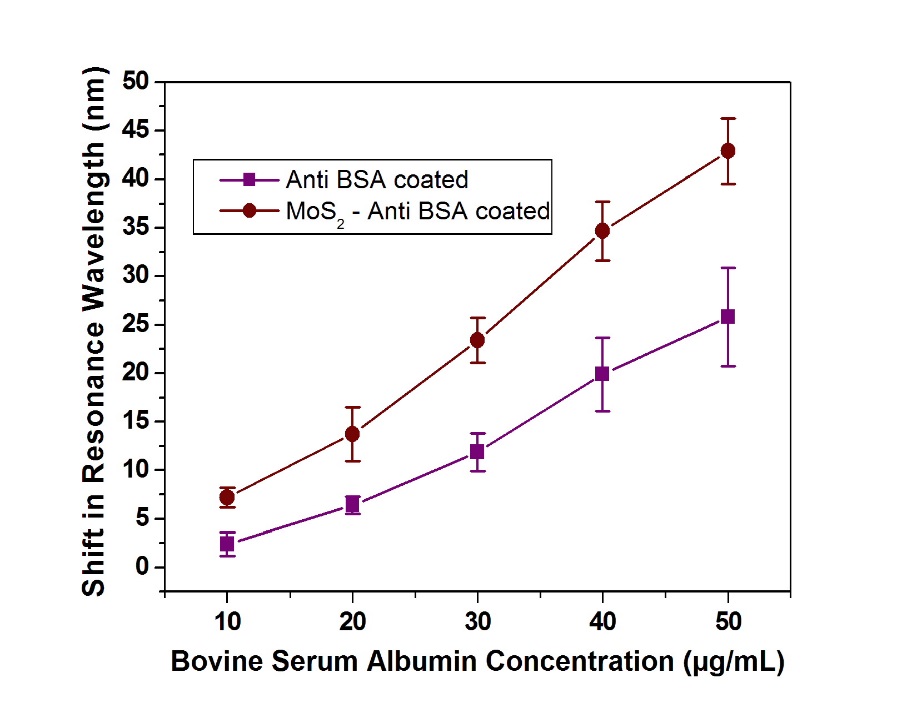


**Fig. S2.** Relative shift in wavelength of conventional SPR sensing probe and developed SPR sensing probe for BSA detection

**Table S1.** Tukey's pairwise comparison of the resonant wavelength in detection of different compounds including BSA, glucose, urea and blank samples

| Group | Mean Diff. | p value |
| --- | --- | --- |
| BSA - Blank | 7.186^*^ | <0.001 |
| BSA - Glucose | 6.471^*^ | <0.001 |
| BSA- Urea | 6.298^*^ | <0.001 |
| Blank - Glucose | -0.715 | 0.095 |
| Blank - Urea | -0.888 | 0.547 |
| Glucose - Urea | 0.173 | 0.547 |

*indicates a significant difference in the resonant wavelength
